# Dietary intake of heat-killed *Lactococcus lactis* H61 delays age-related hearing loss in C57BL/6J mice

**DOI:** 10.1038/srep23556

**Published:** 2016-03-22

**Authors:** Hideaki Oike, Ayako Aoki-Yoshida, Hiromi Kimoto-Nira, Naoko Yamagishi, Satoru Tomita, Yasuyo Sekiyama, Manabu Wakagi, Mutsumi Sakurai, Katsunari Ippoushi, Chise Suzuki, Masuko Kobori

**Affiliations:** 1 National Food Research Institute, National Agriculture and Food Research Organization, 2-1-12 Kannondai, Tsukuba, Ibaraki 305-8642, JAPAN; 2 NARO Institute of Livestock and Grassland Science, National Agriculture and Food Research Organization, 2 Ikenodai, Tsukuba, Ibaraki 305-0901, JAPAN; 3 Graduate school of Agricultural and Life Sciences, The University of Tokyo, 1-1-1 Yayoi, Bunkyo-ku, Tokyo 113-8657, JAPAN

## Abstract

Age-related hearing loss (AHL) is a common disorder associated with aging. In this study, we investigated the effect of the intake of heat-killed *Lactococcus lactis* subsp. *cremoris* H61 (strain H61) on AHL in C57BL/6J mice. Measurement of the auditory brainstem response (ABR) demonstrated that female mice at 9 months of age fed a diet containing 0.05% strain H61 for 6 months maintained a significantly lower ABR threshold than control mice. The age-related loss of neurons and hair cells in the cochlea was suppressed by the intake of strain H61. Faecal analysis of bacterial flora revealed that the intake of strain H61 increased the prevalence of *Lactobacillales*, which is positively correlated with hearing ability in mice. Furthermore, plasma fatty acid levels were negatively correlated with hearing ability. Overall, the results supported that the intake of heat-killed strain H61 for 6 months altered the intestinal flora, affected plasma metabolite levels, including fatty acid levels, and retarded AHL in mice.

Age-related hearing loss (AHL) is a universal feature of mammalian aging and a common sensory disorder in the elderly^1^. In the US, approximately one-third of the population between 65 and 74 years of age experiences hearing loss, and nearly 50% of the population over 75 years of age has hearing problems (NIDCD: http://www.nidcd.nih.gov/health/hearing/Pages/Age-Related-Hearing-Loss.aspx). Experimental mouse models also exhibit AHL under normal housing conditions, and the onset of AHL and intrinsic hearing ability is different among different strains^2^,^3^. Therefore, several studies have investigated the genetics and molecular mechanism of AHL using mouse models.

AHL exhibits an age-dependent decline of hearing function correlated with the loss of spiral ganglion (SG) neurons and sensory hair cells in the cochlea of the inner ear^1^. Loss of SG neurons and hair cells leads to a progressive loss of hearing ability, because these post-mitotic cells do not regenerate in mammals. AHL progresses from high-frequency sounds to low-frequency sounds during aging and is accompanied by the loss of SG neurons and hair cells, beginning from the basal region toward the apical region of the cochlea^4^. Mitochondrial oxidation is thought to be a major cause of cell death in the cochlea, because overexpression of catalase in the mitochondria, but not in the nucleus or cytosol, suppresses the loss of SG neurons and hair cells and prevents AHL^5^. Although there is no medication to restore AHL, dietary supplementation of mitochondrial antioxidants, such as coenzyme Q10, α-lipoic acid, and *N*-acetyl-l-cysteine, retards AHL in C57BL/6J mice^5^. Therefore, prevention of AHL by dietary supplementation is preferable in order to maintain hearing ability.

Probiotic bacteria are living microorganisms that confer health benefits to the host, when administered in adequate amounts. Several probiotic strains have beneficial effects on health such as reduction of serum cholesterol levels, anticarcinogenic effects, improvement of intestinal flora, immunomodulatory effects, alleviation of infections, and extension of lifespan^6^,^7^,^8^,^9^. Lactic acid bacteria are typical probiotics. *Lactococcus lactis* subsp. *cremoris* H61 (strain H61), which has been used for the last 60 years in Japan to produce fermented dairy products, is one of the beneficial strains of lactic acid bacteria^10^. We previously reported that long-term oral intake of a diet containing 0.05% heat-killed strain H61 suppressed the age-associated incidence of skin ulcers, hair loss, and reduction of bone density and body weight in senescence-accelerated mice^10^. In a previous study, the intake of heat-killed strain H61 for 8 weeks improved skin hydration in the forearms and increased the self-evaluation scores for the apparent number of hair follicles and throat dryness in women^11^. Furthermore, the intake of milk fermented by living strain H61 for 4 weeks increased skin hydration and sebum content in young women^12^ or increased the self-evaluation scores for skin elasticity and texture in middle-aged women^13^. However, the mechanisms underlying the beneficial effects of lactic acid bacteria, including strain H61, on aging are largely unknown.

In this study, we hypothesized that the long-term intake of strain H61 contributes to the prevention of AHL. To explore this hypothesis, we examined the effect of the intake of heat-killed strain H61 on AHL in C57BL/6J mice. Furthermore, we analysed the intestinal flora and plasma metabolites to reveal the mechanism underlying the beneficial effects of strain H61.

## Materials and Methods

### Animals and Diets

Animals were handled according to the guidelines of the Ministry of Agriculture, Forestry and Fisheries for laboratory animal studies, and the study was reviewed and approved by the Animal Care and Use Committee of the National Food Research Institute (approval ID: H25-042).

Mice of the popular experimental strain C57BL/6J (male and female, 2 months of age) were obtained from the Institute for Animal Reproduction, Charles River, Japan. The mice were housed at 25 ± 1 °C, 50 ± 5% humidity, and a 12-h light-dark photocycle and had ad libitum access to water and a standard diet (MF; Oriental Yeast, Tokyo, Japan) with or without 0.05% heat-killed strain H61 (The National Institute of Agrobiological Sciences Genebank, Tsukuba, Japan), a concentration that was used in a previous study^10^. The mice were provided the test diet at 3 months of age after 1 month of acclimation. Strain H61 was cultured in MRS broth (BD Biosciences, Franklin Lakes, NJ, USA) by subculturing 1% inoculum overnight at 30 °C. The bacterial cells were harvested and washed once with 0.85% NaCl and then resuspended in distilled water. Heat-killed cells were prepared by treatment at 100 °C for 30 min, followed by centrifugation and lyophilisation.

### Measurement of Auditory Brainstem Response (ABR)

At 9 months of age, ABRs were measured with a tone burst stimulus at 8 kHz and 16 kHz using TDT System 3 equipped with BioSigRP (Tucker Davis Technologies, Alachua, FL, USA) as described previously^14^,^15^. Mice were anesthetised with sodium pentobarbital (70 mg kg^−1^, i.p.; Kyoritsu Seiyaku, Tokyo, Japan), and the needle electrodes were placed subcutaneously at the vertex (active electrode), beneath the pinna of the left ear (reference electrode), and beneath the right ear (ground). The sound stimulus consisted of a 5-ms tone burst, with a rise-fall time of 1.5 ms at frequencies of 8 kHz and 16 kHz. The responses to 500 sweeps were averaged at each intensity level (5-dB steps) to assess the threshold. Hearing threshold was defined as the lowest stimulus intensity that produced reliable peaks in ABR waveforms.

### Cochlea Histology and Survival Cell Counting

The mice were deeply anesthetised with sodium pentobarbital, and blood was collected from the inferior vena cava. The animals were then sacrificed by cervical dislocation, and the temporal bone was excised from the head and divided into cochlear and vestibular parts as described previously^5^. The inner ear, including the cochlea, was excised, and some of the samples were frozen immediately for quantitative reverse transcription-polymerase chain reaction (qRT-PCR) analysis, while the rest were immersed in a fixative containing 4% formaldehyde (Wako Pure Chemical Industries, Osaka, Japan) in phosphate-buffered saline for 24 h. The samples were then decalcified in 10% ethylenediaminetetraacetic acid for more than 1 week. The paraffin-embedded specimens were sliced into 4-μm sections, mounted on glass slides, stained with haematoxylin and eosin, and observed under a light microscope. The Rosenthal’s canal was divided into three regions, apical, middle, and basal, which were used to evaluate cochlear histology. Four mice (two males and two females) per feeding group were used for histopathological assessment. In each mouse, we evaluated every third modiolar section obtained from one unilateral cochlea for a total of nine sections. Tissues from the same animals were used for neuron counting and hair cell counting.

SG neurons were counted in the middle and basal regions of the cochlear sections using a 20× objective as described previously^5^. The corresponding area of the Rosenthal’s canal was measured on digital photomicrographs using Image J 1.47, (National Institutes of Health, Bethesda, MD, USA). The numbers of neurons were expressed as the number of neurons per mm^2^. Nine sections of the unilateral apical, middle, and basal turns were evaluated in one cochlea per mouse.

Outer hair (OH) cells were counted in the middle and basal regions of the cochlear sections using a 40× objective. Hair cells were identified by the presence of a nucleus. The OH cell survival percentage was calculated as the number of intact OH cells present out of three OH cells observed in each turn of one cochlea in tissue sections of mice with normal hearing. Nine sections of the unilateral apical, middle, and basal turns were evaluated in one cochlea per mouse.

### RNA Preparation and qRT-PCR Analysis

Frozen inner ear samples were immersed in RNAlater^®^ solution (Life Technologies, Carlsbad, CA, USA) and stored at −20 °C for three days. Then, the cochlea was excised under a stereoscopic microscope. Total RNA was prepared from the cochlea using TRIzol^®^ RNA isolation reagent (Life Technologies), and cDNA was synthesised using SuperScript™ II reverse transcriptase (Life Technologies) with random hexamers. The transcripts were quantified using ABI Prism 7000 Genetic Analyser (Life Technologies) with Power SYBR^®^ Green Master Mix (Life Technologies) and the following gene-specific primers: 5′-ctaaggccaaccgtgaaaag-3′ and 5′-accagaggcatacagggaca-3′ for *Actb*, 5′-ggaatgcctacgaactcttca-3′ and 5′-ccagctgatgccactcttaaa-3′ for *Bak-1*, 5′-gtgagcggctgcttgtct-3′ and 5′-ggtcccgaagtaggagagga-3′ for *Bax*, 5′-agttgacggaccccaaaag-3′ and 5′-agctggatgctctcatcagg-3′ for *IL1b*, 5′-tgggatctgtcatcgtgct-3′ and 5′-atcaccatgtttctcttgatcg-3′ for *IL17ar*, and 5′-tggagcaacatgtggaactc-3′ and 5′-gtcagcagccggttacca-3′ for *Tgfb1*. The relative amount of each transcript was normalised to the amount of Actb transcript in the same cDNA.

### Analysis of Faecal Flora

Faeces were collected from mice fed a strain H61-containing or control diet for 6 months and stored at −80 °C until further analysis. DNA was extracted from the faeces using the QIAamp^®^ DNA Stool Mini Kit (Qiagen, Hilden, Germany). Pyrosequencing of 16S ribosomal RNA genes was performed by Macrogen Inc. (Seoul, South Korea). The V1–V3 regions of 16S ribosomal RNA genes were amplified from faecal DNA samples by PCR using barcoded fusion primers (27F/518R primer). PCR products were purified using Agencourt^®^ AMPure beads (Beckman Coulter Inc., Brea, CA, USA). All amplicons were then pooled at equimolar ratios into one mixture for pyrosequencing. Emulsion PCR and sequencing were performed according to the manufacturer’s instructions (GS-FLX 454 Titanium; Roche, Basel, Switzerland). The sorted reads corresponding to faecal samples from 20 mice were 90,189 with an average read length of 427 bases. The nucleotide sequence data were submitted to the DDBJ Sequenced Read Archive under the BioSample accession numbers SAMD00042789–SAMD00042808. All the sequences were clustered into the Operational Taxonomic Unit (OTU) to 97% sequence similarity using a 97% identity threshold and CD-HIT-OTU^16^. The OTUs were classified from phylum to genus using the SILVA rRNA database.

### Non-targeted Nuclear Magnetic Resonance (NMR)-based Metabolic Profiling of Plasma Analysis

Blood samples were collected from the inferior vena cava using a heparin-containing syringe and then, separated into plasma and cellular fractions by centrifugation. A total of 50 μl of plasma was mixed with 200 μl of 200 mM potassium phosphate buffer (pH/pD 7.0) in deuterium oxide (D_2_O, 99.9%, Cambridge Isotope Laboratories, Tewksbury, MA, USA). The solution was heated for 5 min at 90 °C with shaking at 1,400 rpm using a ThermoMixer^®^ Comfort (Eppendorf, Hamburg, Germany) and subsequently centrifuged at 21,500 × *g* for 5 min at room temperature. The supernatant was collected and diluted with the same volume of D_2_O containing 2 mM 2,2-dimethyl-2-silapentane-5-sulfonate sodium salt (DSS, Sigma-Aldrich, St. Louis, MO, USA). For NMR-based metabolic profiling, the ^1^H NMR spectrum was recorded in 5.0-mm O.D. × 103.5-mm NMR tubes (Norell, Landisville, NJ, USA) on an Avance-500 spectrometer equipped with a CryoProbe and a SampleJet (Bruker BioSpin, Ettlingen, Germany), with a proton frequency of 500.23 MHz at 298 K as described previously^17^. To remove broad signals from macromolecules and selectively highlight small molecule metabolites, the Carr-Purcell-Meiboom-Gill (CPMG) spin-echo sequence with presaturation was used employing the Bruker pulse program cpmgpr1d^18^,^19^. The acquisition parameters were as follows: spectral width, 20 ppm; offset frequency, 4.7 ppm; spectral data points, 64 k; proton 90° pulse, 17.5 μs; relaxation delay, 4 s; increment delay, 20 s; number of scans, 256; receiver gain, 406. A 400-μs spin-echo delay and 80 times of loops were applied as described previously^20^. Solvent signal was removed by the pre-saturation method.

Multivariate analysis based on the ^1^H NMR spectra was performed as described previously^17^. Briefly, the ^1^H NMR spectra measured were processed using TopSpin 3.2 (Bruker BioSpin) and subdivided into each 0.04-ppm integrated regions (buckets) in the spectral range of 10.00–0.50 ppm using Amix 3.9.14 (Bruker BioSpin). Twenty buckets belonging to 5.20–4.40 ppm were excluded, because they contained residual water signals, resulting in a dataset of 218 buckets. For normalizing the buckets, the total intensity option provided by Amix was applied, because the signal intensity of the internal standard (DSS) was inconsistent among the plasma samples due to its interaction with serum albumin^21^,^22^. The generated dataset was then appended with the ABR values and used for multivariate analysis. Partial least squared (PLS) regression was performed using SIMCA 13.0.3.0 (Umetrics, Umea, Sweden). Pareto scaling was applied to the NMR bucket data, whereas mean centring was used for the ABR values. PLS models were evaluated by leave-one-out cross-validation.

For metabolite identification, the ^1^H-^13^C heteronuclear single quantum coherence (HSQC) spectrum was measured for representative samples on the same instrument with a carbon frequency of 125.78 MHz, applying the following parameters: 90° pulse values, 17.5 μs (proton) and 15 μs (carbon); relaxation delay, 2 s; spectral width, 130 ppm (f1) and 12 ppm (f2); offset frequency, 70 ppm (f1) and 5.5 ppm (f2); data points, 256 increments of 2 k; scans, 256. The spectrum was processed with NMRPipe and analysed using NMRDraw^23^ to generate a peak table; metabolite annotation was performed by applying the peak table to a SpinAssign program through the PRIMe web service (Platform for RIKEN Metabolomics; http://prime.psc.riken.jp/)^24^. Other public databases, such as the Human Metabolomics Database (http://www.hmdb.ca/)^25^ and the Biological Magnetic Resonance Data Bank (http://www.bmrb.wisc.edu/)^26^ were also referred. Other 2D NMR spectra, including the double quantum filtered correlated spectroscopy (DQF-COSY) spectrum, total correlation spectroscopy (TOCSY) spectrum, and ^1^H-^13^C heteronuclear multiple-bond connectivity (HMBC) spectrum, were also recorded to facilitate metabolite annotation.

### Measurement of Non-esterified Fatty Acids (NEFAs)

Blood NEFA was measured using a LabAssay™ NEFA kit (ACS-ACOD method; Wako Pure Chemical Industries) according to the manufacturer’s instructions.

### Statistical Analysis

All statistical analyses were carried out using R 3.2.0 (http://www.R-project.org/) or SIMCA 13.0.3.0. Data were expressed as mean values ± standard error (SE). A *p*-value of less than 0.05 was considered statistically significant.

## Results

### Intake of Strain H61 Retards the Onset of AHL in C57BL/6J mice

**Figure 1:**
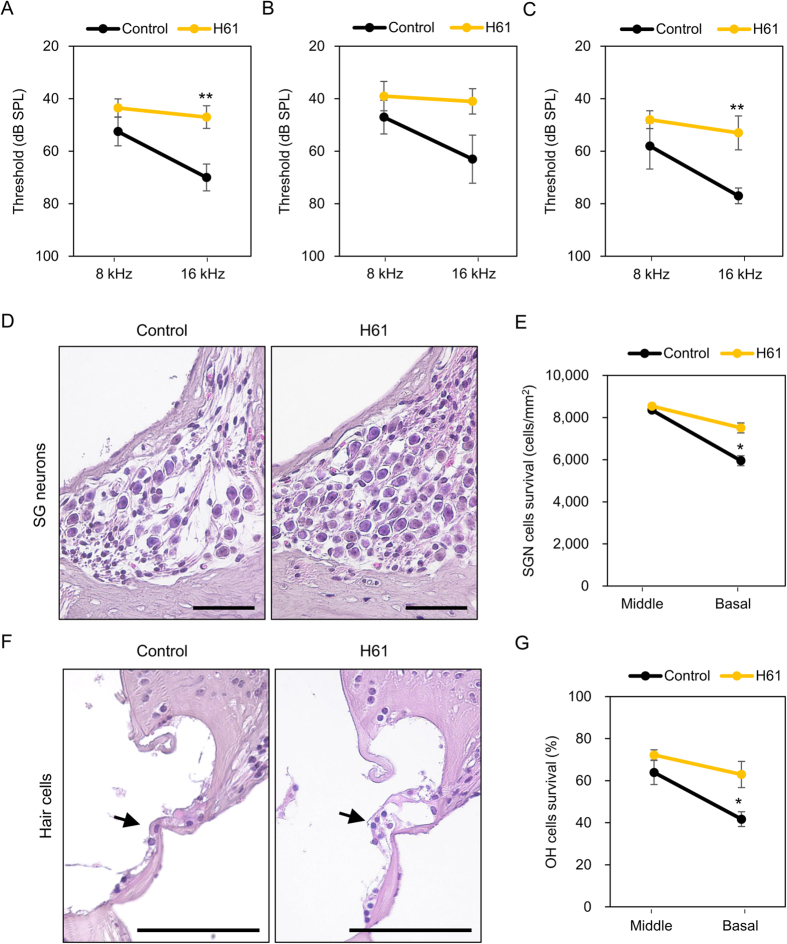
*Lactococcus lactis* strain H61 retards the onset of age-related hearing loss (AHL) and protects cell death in the cochlea in C57BL/6J mice. (**A–C**) Auditory brainstem response (ABR) hearing thresholds were measured at 8 kHz and 16 kHz in mice at 9 months of age fed on a diet containing strain H61 for 6 months and in control mice of the same age. Each panel shows the results obtained for males ((**B**) n = 5), females ((**C**) n = 5), or both ((**A**) n = 10) **(D–G**) Histological sections containing spiral ganglion (SG) neurons (**D**) or hair cells (**F**) in the basal area of the cochlea (Scale bar, 50 μm). Arrows indicate the outer hair (OH) cells. Density of SG neurons (**E**) or survival rate of OH cells (**G**) in the basal and middle cochlear regions was counted. Mean ± standard error, *significance at *p* < 0.05, **significance at *p* < 0.01.

To elucidate the effect of the intake of strain H61 on AHL in mice, we quantified ABR thresholds in C57BL/6J mice fed on a diet containing 0.05% heat-killed strain H61 or a control diet for 6 months. The average ABR threshold at 16 kHz in the strain H61-fed mice was 47 ± 4 dB SPL (Fig. 1A; n = 10). This value was significantly lower (better hearing ability) than the threshold of control mice (70 ± 5 dB SPL; n = 10, *p* = 0.003 by *t*-test). Although the intake of strain H61 maintained a lower ABR threshold at 16 kHz compared with control mice in both genders, the statistical differences were more significant in female mice (Fig. 1B and C; *p* = 0.067 and *p* = 0.0097 in males and females, respectively; n = 5). The ABR thresholds at 8 kHz were lower and not significantly different in the two feeding groups (53 ± 5 and 44 ± 3 dB SPL in control and H61 groups, respectively, n = 10, *p* = 0.18 by *t*-test), indicating that AHL did not develop at 8 kHz in mice at 9 months of age. These results indicated that the intake of strain H61 retarded AHL, more profoundly in females than males, especially at 16 kHz. Therefore, we used 16 kHz as the threshold for the subsequent correlation analyses between hearing ability and faecal flora or plasma metabolites.

To confirm the histological relevance of the differences in ABR thresholds between the feeding groups, the numbers of surviving SG neurons and sensory hair cells in the cochlea sections were counted (Fig. 1D–G). The survival of SG neurons and OH cells in the basal area of the cochlea, which detects higher frequency sound, was significantly increased in mice fed a diet containing strain H61 (Fig. 1E,G). However, the survival numbers of SG neurons and OH cells were higher in the middle region than in the basal region of the cochlea, and the values did not differ between the feeding groups. These results were consistent with those of the hearing test and showed that AHL starts with high-frequency sound but does not progress with low-frequency sound.

Since the apoptosis of SG neurons and hair cells in the inner ear is mediated by *Bak1*, but not *Bax*^5^, we examined the mRNA expression levels of these two genes by qRT-PCR. The expression of *Bak1* was significantly downregulated in the H61 group (n = 8–10, *p* = 0.021 by *t*-test), whereas *Bax* was expressed at equivalent levels in both feeding groups (Supplementary Figure 1A; n = 8–10, *p* = 0.59). These results suggested that the intake of strain H61 protected against the *Bak-*dependent apoptosis of SG neurons and hair cells in the cochlea.

### Intake of Strain H61 Alters Bacterial Flora

**Figure 2:**
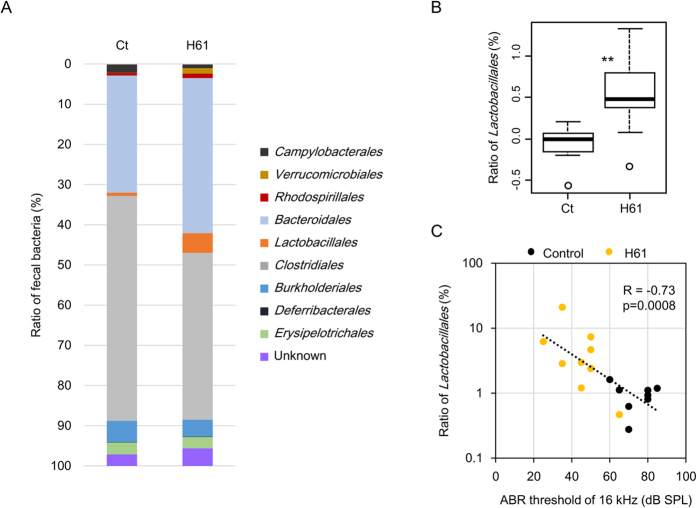
Intake of *Lactococcus lactis* strain H61 alters faecal flora in mice. (**A**) Average compositions of bacterial flora in mice at 9 months of age fed on a diet containing strain H61 for 6 months and in control mice of the same age (n = 10). (**B**) Ratio of *Lactobacillales* (Log 2 values, mean ± SE, n = 8–9, **p < 0.01) in the faeces. (**C)** Correlation between the ratio of *Lactobacillales* and the auditory brainstem response (ABR) hearing threshold at 16 kHz (n = 17).

We hypothesised that the intake of strain H61 affected the gut flora and humoral factors in the blood, leading to retarded AHL. Therefore, we analysed bacterial 16S ribosomal RNA gene sequences in the faeces to examine changes in the gut flora. The results revealed that the bacterial ratios in the faeces were altered by the intake of heat-killed strain H61 (Fig. 2A). Only the prevalence of *Lactobacillales* was significantly altered in the strain H61-fed group (Fig. 2B; n = 8–9, *p* = 0.0063 by *t*-test). Additionally, the ratio of *Lactobacillales* was positively correlated with hearing ability, defined as the ABR threshold at 16 kHz (Fig. 2C; n = 17, R = −0.73, *p* = 0.0008). The genus *Lactobacillus* consists of four OTUs; however, a single OTU, *Lactobacillus johnsonii*, mainly contributed to the increase of *Lactobacillales* and positive correlation with the ABR threshold (Supplementary Figure 2A,B; n = 15, R = −0.47, *p* = 0.056). Moreover, two other OTUs, classified as *Bacteroidetes* (n = 18, R = −0.50, *p* = 0.025) and unknown phylum (n = 19, R = −0.67, *p* = 0.0017), showed a positive correlation with hearing ability at 16 kHz (Supplementary Figure 2B).

### Non-targeted NMR-based Metabolic Profiling of Plasma

**Figure 3:**
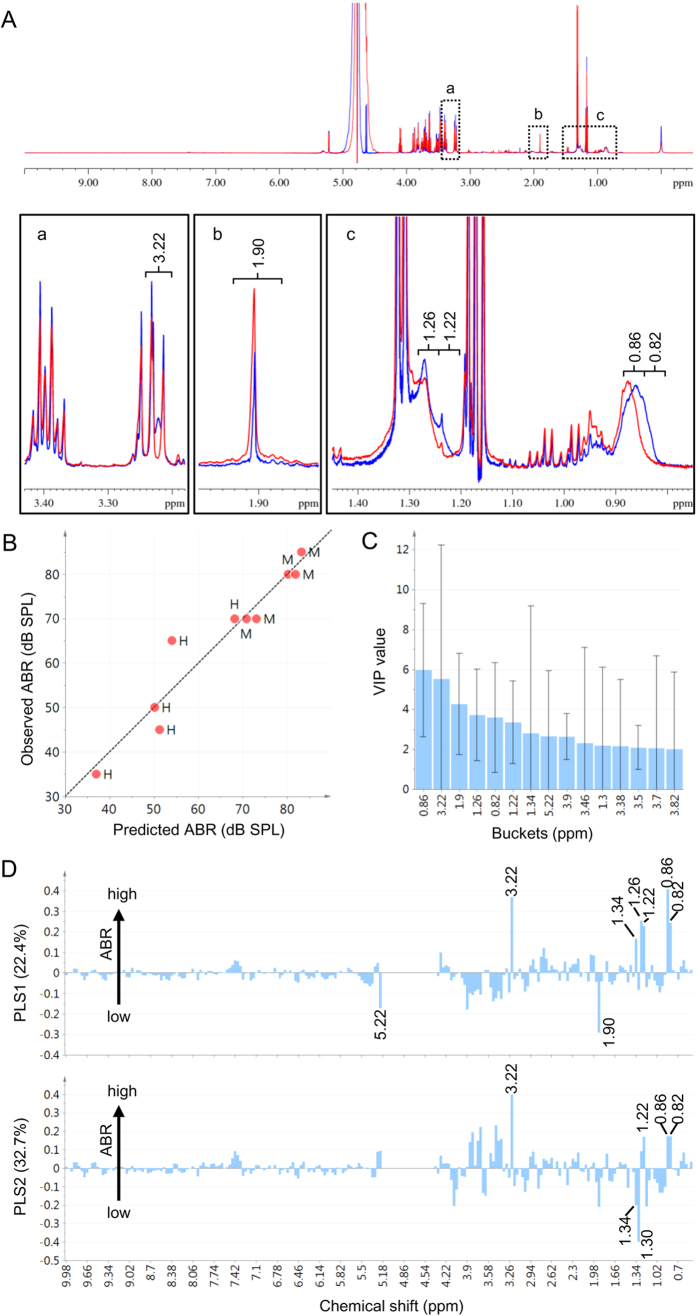
Nuclear magnetic resonance (NMR)-based metabolome analysis of plasma samples from female mice at 9 months of age fed on a diet containing *Lactococcus lactis* strain H61 for 6 months and from control female mice of the same age. (**A**) ^1^H NMR spectral comparison between representative female mice fed on strain H61 (red) and control female mice (blue). The whole spectrum is shown in the top panel, and magnified regions (a–c) are shown below. For comparison of signal intensities, the spectra were recorded by applying the same acquisition conditions, including receiver gain, number of scans, and relaxation delay; the two spectra are overlaid as are. Spectral ranges of the top six buckets with the highest variable importance in the projection (VIP) scores (shown in (**C)**) are indicated over the magnified spectra. (**B–D**) Partial least squared (PLS) regression based on the ABR threshold at 16 kHz and metabolic profiling; observed versus predicted plot of auditory brainstem response ((**B**) n = 5, M: control, H: H61), variable importance plot (**C**), and loading plot of PLS1 and PLS2 (**D**).

Next, we performed a metabolome analysis on plasma samples using ^1^H NMR to identify metabolites that are quantitatively affected by the intake of strain H61. We used blood samples from strain H61-fed and control female mice (n = 5 for each feeding group), because strain H61 significantly retarded AHL in this gender (Fig. 1B,C). As expected, the ^1^H NMR spectra showed some differences between the feeding groups (Fig. 3A). The following metabolites were predominantly detected: glucose (Glc), lactate (LA), ethanol (EtOH), acetate (HOAc), citrate (CA), alanine (Ala), glutamine (Gln), lysine (Lys), valine (Val), leucine (Leu), isoleucine (Ile), glycine (Gly), creatine, and taurine (Supplementary Figure 3). Signals from the –CH3 and –CH2– moieties of the fatty acid groups (FAGs) of lipid compounds, including both saturated and unsaturated chains, were also observed.

To identify the responsible metabolites for the changes in ABR values, PLS regression was performed based on the dataset generated from the ^1^H NMR spectrum. The resulting model suggested a strong correlation between the ABR value and the spectrum (Fig. 3B). The first two latent variables (PLS1 and PLS2) that were obtained from the PLS model provided a cumulative determination coefficient (R^2^) of 0.93 with a cumulative cross-validation determination coefficient (Q^2^) of 0.52. PLS1 and PLS2 explained 22% and 33% of the total variance, respectively. The variable importance in the projection (VIP) score plot revealed variables responsible for predicting ABR values and highlighted the importance of buckets 0.82–0.86 (FAG), 3.22 (phosphocholine), 1.90 (HOAc), and 1.22–1.26 (FAG; Fig. 3A,C); however, bucket 3.22 (phosphocholine) had an unacceptable error in the VIP score. The loading plot indicated higher signal intensity in bucket 1.90 (HOAc), which explained the lower ABR threshold for this bucket (better hearing ability), whereas the other buckets contributed to a higher ABR threshold (Fig. 3D).

### Plasma Fatty Acid Levels are Correlated with the ABR Threshold

**Figure 4:**
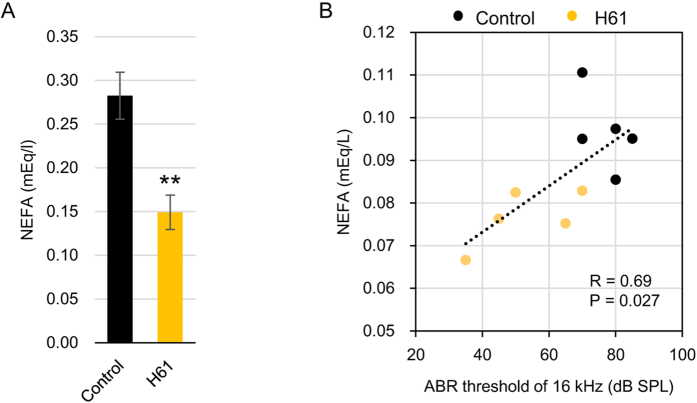
Correlation between plasma non-esterified fatty acid (NEFA) levels and auditory brainstem response (ABR) threshold in female mice. (**A**) Concentration of plasma NEFA in female mice fed on *Lactococcus lactis* strain H61 for 6 months and in female control mice (n = 5, **p < 0.01 by *t*-test). (**B)** Correlation between plasma NEFA levels and the ABR threshold at 16 kHz (n = 10).

The NMR-based metabolome analysis suggested that fatty acids negatively contribute to ABR threshold. Therefore, we measured plasma NEFA levels from female mice using an enzyme-based commercial kit. Plasma NEFA levels were significantly lower in the H61-fed group than in the control group (Fig. 4A; n = 5, *p* = 0.0039 by *t*-test). Furthermore, plasma NEFA levels were negatively correlated with hearing ability at 16 kHz (Fig. 4B; n = 10, R = 0.69, *p* = 0.027), results that were consistent with those of the NMR analysis.

## Discussion

In the present study, we demonstrated that the intake of heat-killed strain H61 retarded AHL in C57BL/6J mice. To our knowledge, this is the first report on the anti-aging effect of the intake of lactic acid bacteria on the hearing system, which might reflect wider changes in the body, as our previous study showed that strain H61 suppressed some symptoms of skin, hair, and bone aging in senescence-accelerated mice^27^. However, these symptoms in skin and bones could not be evaluated in this study, because C57BL/6J mice at 9 months of age were too young to show any signs of aging. We also found that the intake of strain H61 altered the bacterial flora in the faeces, and that the ratio of *Lactobacillaceae* was correlated with hearing ability. Moreover, non-biased metabolome analysis of the blood by ^1^H NMR indicated a negative correlation between plasma fatty acid levels and hearing ability. These results suggested that the intake of strain H61 alters the gut flora and changes the levels of bacterial products and metabolites in the blood, affecting the oxidative state of the inner ear, reducing the number of apoptotic cells in the cochlea, and leading to retard AHL. However, the direct causes that retard AHL remained to be elucidated, because correlations between hearing ability and floral changes or plasma fatty acids are indirect evidences.

The test diets were administrated to C57BL/6J mice from the age of 3 months to the age of 9 months, because C57BL/6J mice show early onset of ALH, and hearing loss at high-frequency sounds (16–28 kHz) is prominent in middle-aged mice^27^,^28^. Our results demonstrated the significant increase of the ABR threshold at 16 kHz in mice at 9 months of age and the loss of SG neurons and hair cells in the basal cochlea region, indicating the beginning of AHL at this frequency. However, the ABR threshold at 8 kHz was lower, and loss of cochlear cells was not detected in the middle region, suggesting that AHL did not progress at this frequency. The intake of strain H61 significantly improved the AHL at 16 kHz and cochlear cell numbers in the basal area, but did not affect the AHL at 8 kHz and cochlear cell numbers in the middle region, suggesting that strain H61 retarded onset of AHL rather than improving hearing ability non-specifically.

Oral intake of probiotic bacteria retards mammalian age-dependent disorders in many tissues, including intestine, skin, and immune cells, improves memory, and prolongs lifespan in rodents^6^,^29^,^30^,^31^,^32^. These beneficial effects are partly achieved by improving the intestinal environment and suppressing inflammation^6^. Aging is associated with systemic low-grade chronic inflammation and increased levels of cytokines and proinflammatory markers^33^,^34^. As a result, many genes associated with inflammation and immune response are upregulated with age in the mouse cochlea^35^. The intake of heat-killed strain H61 might alter the immune response and reduce inflammation through changing the intestinal flora, since it affects the production of interferon-γ and IL-12 secreted by immune cells in mice^10^. Though, we did not identify any differences in the expression level of inflammation associated genes in the inner ear^35^ (*IL1b*, *IL17ar*, and *Tgfb1*; Supplementary Figure 1B) in the present study, we demonstrated that the intake of strain H61 increased the ratio of *Lactobacillales*, indicating an improvement in the intestinal environment and immune response^36^. *L. johnsonii* has been shown to reduce the prevalence of *Clostridium perfringens* in poultry^37^. The immunomodulatory activity of *Lactobacillales* might reduce chronic inflammation and retard aging of the inner ear indirectly. Notably, the PLS regression analysis indicated that plasma acetate levels positively contributed to AHL retarding. These changes might reflect alterations in gut bacterial composition, because short chain fatty acids (SCFAs), containing acetate, propionate and butyrate moieties, are the major products of anaerobic bacterial fermentation. SCFAs produced by bacteria might retard AHL progression, since they reduce inflammation through the activation of GPCRs (GPR41 and GPR43) or leukocyte function, including the production of cytokines, eicosanoids, and chemokines^38^. SCFAs also affect lipid metabolism and appetite^39^. In this study, the intake of strain H61 reduced plasma NEFA levels, and hearing ability was negatively correlated with plasma NEFA levels. NEFAs are middle or long chain fatty acids; the detection kit used in this study is based on the reaction of a bacterial acyl-CoA oxidase that oxidises carbon chain lengths of 4–20 and is mostly active toward lauroyl-CoA, rather than acetyl- or succinyl-CoA^40^. The intake of strain H61 might affect lipid metabolism and reduce plasma NEFA levels, leading to reduced inflammation.

In summary, we demonstrated that oral supplementation of strain H61 retards AHL in mice. Previous reports have indicated that mitochondrial antioxidants also prevent AHL in middle-aged mice^5^,^41^. Prevention is important in the case of progressive disorders, such as AHL. Lactic acid bacteria have been used for centuries to ferment food, and they possess a wide range of anti-aging effects. Therefore, our findings might aid the development of anti-aging supplements that promote a healthy life style and prevent progressive disorders.

## Additional Information

**How to cite this article**: Oike, H. *et al.* Dietary intake of heat-killed *Lactococcus lactis* H61 delays age-related hearing loss in C57BL/6J mice. *Sci. Rep.* **6**, 23556; doi: 10.1038/srep23556 (2016).

## Supplementary Material

Supplementary Information

## References

[b1] Someya S. & Prolla T. A. Mitochondrial oxidative damage and apoptosis in age-related hearing loss. Mech Ageing Dev 131, 480–486, 10.1016/j.mad.2010.04.006 (2010). PMC4086639 20434479

[b2] Zheng Q. Y., Johnson K. R. & Erway L. C. Assessment of hearing in 80 inbred strains of mice by ABR threshold analyses. Hear Res 130, 94–107 (1999). 10320101 10.1016/s0378-5955(99)00003-9 PMC2855304

[b3] Jones S. M. *et al.* A comparison of vestibular and auditory phenotypes in inbred mouse strains. Brain Res 1091, 40–46, 10.1016/j.brainres.2006.01.066 (2006). 16499890 PMC2859199

[b4] Keithley E. M., Canto C., Zheng Q. Y., Fischel-Ghodsian N. & Johnson K. R. Age-related hearing loss and the ahl locus in mice. Hearing Research 188, 21–28, 10.1016/s0378-5955(03)00365-4 (2004). 14759567 PMC2858220

[b5] Someya S. *et al.* Age-related hearing loss in C57BL/6J mice is mediated by Bak-dependent mitochondrial apoptosis. Proc Natl Acad Sci USA 106, 19432–19437, 10.1073/pnas.0908786106 (2009). 19901338 PMC2780799

[b6] Matsumoto M., Kurihara S., Kibe R., Ashida H. & Benno Y. Longevity in mice is promoted by probiotic-induced suppression of colonic senescence dependent on upregulation of gut bacterial polyamine production. PLoS One 6, e23652, 10.1371/journal.pone.0023652 (2011). 21858192 PMC3156754

[b7] Harrison V. C. & Peat G. Serum cholesterol and bowel flora in the newborn. Am J Clin Nutr 28, 1351–1355 (1975). 45573 10.1093/ajcn/28.12.1351

[b8] Fernandez M., Hudson J. A. & Korpela R. & de los Reyes-Gavilan, C. G. Impact on human health of microorganisms present in fermented dairy products: an overview. Biomed Res Int 2015, 412714, 10.1155/2015/412714 (2015). 25839033 PMC4369881

[b9] Kumar M. *et al.* Cancer-preventing attributes of probiotics: an update. Int J Food Sci Nutr 61, 473–496, 10.3109/09637480903455971 (2010). 20187714

[b10] Kimoto-Nira H. *et al.* Anti-ageing effect of a lactococcal strain: analysis using senescence-accelerated mice. Br J Nutr 98, 1178–1186, 10.1017/S0007114507787469 (2007). 17617939

[b11] Kimoto-Nira H., Aoki R., Sasaki K., Suzuki C. & Mizumachi K. Oral intake of heat-killed cells of *Lactococcus lactis* strain H61 promotes skin health in women. J Nutr Sci 1, e18, 10.1017/jns.2012.22 (2012). 25191547 PMC4153081

[b12] Kimoto-Nira H. *et al.* Effects of ingesting milk fermented by *Lactococcus lactis* H61 on skin health in young women: a randomized double-blind study. J Dairy Sci 97, 5898–5903, 10.3168/jds.2014-7980 (2014). 25022690

[b13] Kimoto-Nira H., Moriya N., Sasaki K. & Suzuki C. Effects of ingesting milk fermented by *Lactococcus Lactis* H61 on skin properties and health biomarkers in middle-aged women: a randomized, double-blind study. Journal of Aging Research & Clinical Practice 4, 109–115 (2015).

[b14] Someya S. *et al.* Sirt3 mediates reduction of oxidative damage and prevention of age-related hearing loss under caloric restriction. Cell 143, 802–812, 10.1016/j.cell.2010.10.002 (2010). 21094524 PMC3018849

[b15] Hardisty-Hughes R. E., Parker A. & Brown S. D. A hearing and vestibular phenotyping pipeline to identify mouse mutants with hearing impairment. Nat Protoc 5, 177–190, 10.1038/nprot.2009.204 (2010). 20057387

[b16] Huang Y., Niu B., Gao Y., Fu L. & Li W. CD-HIT Suite: a web server for clustering and comparing biological sequences. Bioinformatics 26, 680–682, 10.1093/bioinformatics/btq003 (2010). 20053844 PMC2828112

[b17] Tomita S. *et al.* A NMR-based, non-targeted multistep metabolic profiling revealed L-rhamnitol as a metabolite that characterised apples from different geographic origins. Food Chem 174, 163–172, 10.1016/j.foodchem.2014.11.028 (2015). 25529666

[b18] Nicholson J. K., Foxall P. J., Spraul M., Farrant R. D. & Lindon J. C. 750 MHz ^1^H and ^1^H-^13^C NMR spectroscopy of human blood plasma. Anal Chem 67, 793–811 (1995). 7762816 10.1021/ac00101a004

[b19] Meiboom S. & Gill D. Modified Spin-Echo Method for Measuring Nuclear Relaxation Times. Review of Scientific Instruments 29, 688, 10.1063/1.1716296 (1958).

[b20] Beckonert O. *et al.* Metabolic profiling, metabolomic and metabonomic procedures for NMR spectroscopy of urine, plasma, serum and tissue extracts. Nat Protoc 2, 2692–2703, 10.1038/nprot.2007.376 (2007). 18007604

[b21] de Graaf R. A. & Behar K. L. Quantitative ^1^H NMR spectroscopy of blood plasma metabolites. Anal Chem 75, 2100–2104, 10.1021/ac020782+ (2003). 12720347

[b22] Nowick J. S., Khakshoor O., Hashemzadeh M. & Brower J. O. DSA: a new internal standard for NMR studies in aqueous solution. Org Lett 5, 3511–3513, 10.1021/ol035347w (2003). 12967312

[b23] Delaglio F. *et al.* NMRPipe: a multidimensional spectral processing system based on UNIX pipes. J Biomol NMR 6, 277–293 (1995). 8520220 10.1007/BF00197809

[b24] Akiyama K. *et al.* PRIMe: a Web site that assembles tools for metabolomics and transcriptomics. In Silico Biol 8, 339–345 (2008). 19032166

[b25] Wishart D. S. *et al.* HMDB: the Human Metabolome Database. Nucleic Acids Res 35, D521–526, 10.1093/nar/gkl923 (2007). 17202168 PMC1899095

[b26] Ulrich E. L. *et al.* BioMagResBank. Nucleic Acids Res 36, D402–408, 10.1093/nar/gkm957 (2008). 17984079 PMC2238925

[b27] Vlajkovic S. M. *et al.* Adenosine kinase inhibition in the cochlea delays the onset of age-related hearing loss. Exp Gerontol 46, 905–914, 10.1016/j.exger.2011.08.001 (2011). 21846498 PMC3200489

[b28] Kane K. L. *et al.* Genetic background effects on age-related hearing loss associated with Cdh23 variants in mice. Hear Res 283, 80–88, 10.1016/j.heares.2011.11.007 (2012). 22138310 PMC3277672

[b29] Gill H. S., Rutherfurd K. J. & Cross M. L. Dietary probiotic supplementation enhances natural killer cell activity in the elderly: an investigation of age-related immunological changes. J Clin Immunol 21, 264–271 (2001). 11506196 10.1023/a:1010979225018

[b30] Jeong J. J. *et al.* Probiotic Mixture KF Attenuates Age-Dependent Memory Deficit and Lipidemia in Fischer 344 Rats. J Microbiol Biotechnol 25, 1532–1536, 10.4014/jmb.1505.05002 (2015). 25975611

[b31] Woo J. Y. *et al.* *Lactobacillus pentosus var. plantarum* C29 ameliorates memory impairment and inflammaging in a D-galactose-induced accelerated aging mouse model. Anaerobe 27, 22–26, 10.1016/j.anaerobe.2014.03.003 (2014). 24657159

[b32] Jeong J. H., Lee C. Y. & Chung D. K. Probiotic Lactic Acid Bacteria and Skin Health. Crit Rev Food Sci Nutr, 0, 10.1080/10408398.2013.834874 (2015). 26287529

[b33] Michaud M. *et al.* Proinflammatory Cytokines, Aging, and Age-Related Diseases. J Am Med Dir Assoc 14, 877–882, 10.1016/j.jamda.2013.05.009 (2013). 23792036

[b34] Guarner V. & Rubio-Ruiz M. E. Low-grade systemic inflammation connects aging, metabolic syndrome and cardiovascular disease. Interdiscip Top Gerontol 40, 99–106, 10.1159/000364934 (2015). 25341516

[b35] Someya S., Yamasoba T., Prolla T. A. & Tanokura M. Genes encoding mitochondrial respiratory chain components are profoundly down-regulated with aging in the cochlea of DBA/2J mice. Brain Res 1182, 26–33, 10.1016/j.brainres.2007.08.090 (2007). 17964557

[b36] Turpin W., Humblot C., Thomas M. & Guyot J. P. *Lactobacilli* as multifaceted probiotics with poorly disclosed molecular mechanisms. Int J Food Microbiol 143, 87–102, 10.1016/j.ijfoodmicro.2010.07.032 (2010). 20801536

[b37] La Ragione R. M., Narbad A., Gasson M. J. & Woodward M. J. *In vivo* characterization of *Lactobacillus johnsonii* FI9785 for use as a defined competitive exclusion agent against bacterial pathogens in poultry. Letters in Applied Microbiology 38, 197–205, 10.1111/j.1472-765X.2004.01474.x (2004). 14962040

[b38] Vinolo M. A., Rodrigues H. G., Nachbar R. T. & Curi R. Regulation of inflammation by short chain fatty acids. Nutrients 3, 858–876, 10.3390/nu3100858 (2011). 22254083 PMC3257741

[b39] Byrne C. S., Chambers E. S., Morrison D. J. & Frost G. The role of short chain fatty acids in appetite regulation and energy homeostasis. Int J Obes (Lond) 39, 1331–1338, 10.1038/ijo.2015.84 (2015). 25971927 PMC4564526

[b40] Shimizu S., Yasui K., Tani Y. & Yamada H. Acyl-CoA oxidase from Candida tropicalis. Biochem Biophys Res Commun 91, 108–113 (1979). 518611 10.1016/0006-291x(79)90589-8

[b41] Tian G. *et al.* Ubiquinol-10 supplementation activates mitochondria functions to decelerate senescence in senescence-accelerated mice. Antioxid Redox Signal 20, 2606–2620, 10.1089/ars.2013.5406 (2014). 24124769 PMC4025630

