# Supplementary material for: Dietary intake of heat-killed Lactococcus lactis H61 delays age-related hearing loss in C57BL/6J mice
Source: Sci Rep. 2016 Mar 22;6:23556. doi: 10.1038/srep23556 (PMC4802309; doi:10.1038/srep23556)
Supplement: Supplementary Information [file srep23556-s1.doc]

**Title: Dietary intake of heat-killed *Lactococcus lactis* H61 prevents age-related hearing loss in C57BL/6J mice**

**Authors: Hideaki Oike^1, *^, Ayako Aoki-Yoshida^2, 3^, Hiromi Kimoto-Nira^2^, Naoko Yamagishi^2^, Satoru Tomita^1^, Yasuyo Sekiyama^1^, Manabu Wakagi^1^, Mutsumi Sakurai^1^, Katsunari Ippoushi^1^, Chise Suzuki^2^, and Masuko Kobori^1^**

^1^National Food Research Institute, National Agriculture and Food Research Organization, 2-1-12 Kannondai, Tsukuba, Ibaraki 305-8642, JAPAN

^2^NARO Institute of Livestock and Grassland Science, National Agriculture and Food Research Organization, 2 Ikenodai, Tsukuba, Ibaraki 305-0901, JAPAN

^3^Graduate school of Agricultural and Life Sciences, The University of Tokyo, 1-1-1 Yayoi, Bunkyo-ku, Tokyo 113-8657, JAPAN

*correspondence: oike@affrc.go.jp

**Supplementary Fig. 1 Expressions of apoptotic genes (A) and inflammatory genes (B) in cochlea**

Expression levels of the two apoptotic genes (A) and three inflammatory genes (B) in the cochlea. Total RNA was extracted from the cochlea of mice fed on a diet containing strain H61 or not using TRIzol® reagent (Life Technologies). mRNAs were quantified by qRT-PCR using Power SYBR® Green Master Mix (Life Technologies) with gene specific primers. The relative amount of each transcript was normalised to the amount of Actb transcripts in the same cDNA. Values are presented as means ± SE (n = 8–10 containing both sexes; **p* < 0.05, *t*-test).

**Supplementary Fig. 2** **OTU details in *Lactobacillus***

OTUs in *Lactobacillus*. Four OTUs were detected in the genus *Lactobacillus* by analysis of faecal flora; the actual read count for each sample is shown (A). Correlation between the counts of OTU-2, 4 and 6 and hearing ability measured as ABR threshold at 16 kHz (B).

**Supplementary Fig. 3** **Metabolite annotation of representative ^1^H NMR spectra of plasma sample**

Metabolite annotation of a representative ^1^H-NMR spectrum of a plasma sample from a control mouse. Abbreviations: Glc, glucose; LA, lactate; EtOH, ethanol; HOAc, acetate; CA, citrate; Ala, alanine; Gln, glutamine; Lys, lysine; Val, valine; Leu, leucine; Ile, isoleucine; FAG, fatty acid groups; DSS, 2,2-dimethyl-2-silapentane-5-sulfonate (internal standard). Signal intensity was adjusted for each spectral region displayed.
